# Targeting Human *β*-Microglobulin with Monoclonal Antibodies in Multiple Myeloma - A Potential in Treatment

**DOI:** 10.4172/2167-7700.1000190

**Published:** 2016-02-20

**Authors:** Mingjun Zhang, Jin He, Jing Yang

**Affiliations:** 1 Department of Lymphoma/Myeloma, Division of Cancer Medicine, The University of Texas MD Anderson Cancer Center, Houston, Texas, USA; 2 Department of Cancer Biology, Lerner Research Institute, Cleveland Clinic, USA; 3 Cancer Research Institution, Guangzhou Medical University, Guangzhou, China

**Keywords:** Monoclonal, Antibodies, Neoplasm, Immunotherapy, Multiple myeloma

## Introduction

Multiple myeloma (MM) is a clonal plasma cell neoplasm that utilizes bone marrow microenvironment for survival and proliferation [1-3]. However, current therapies could rarely cure MM. The relapse or refractory aspect of the disease is commonly seen in MM patients, especially among patients with high-risk MM. In past decades, targeted immunotherapy with monoclonal antibodies (mAbs) emerged as a major new treatment modality that offered great benefits for MM patients [4]. Different approaches, aimed at finding potential mAb-based therapeutics for this disease including identification of alternative, or novel, target antigens [5], conjugation of mAbs with classic or novel drugs [6], and generation of chimeric antigen receptor T cells with specific mAbs [7], have been developed by scientists. Recently, our group has generated the mAbs that work directly against human β2-microglobulin (β2M) both in vitro and in the mouse experiments, and has demonstrated that β2M is a potential target for MM treatment [8].

**Figure 1:**
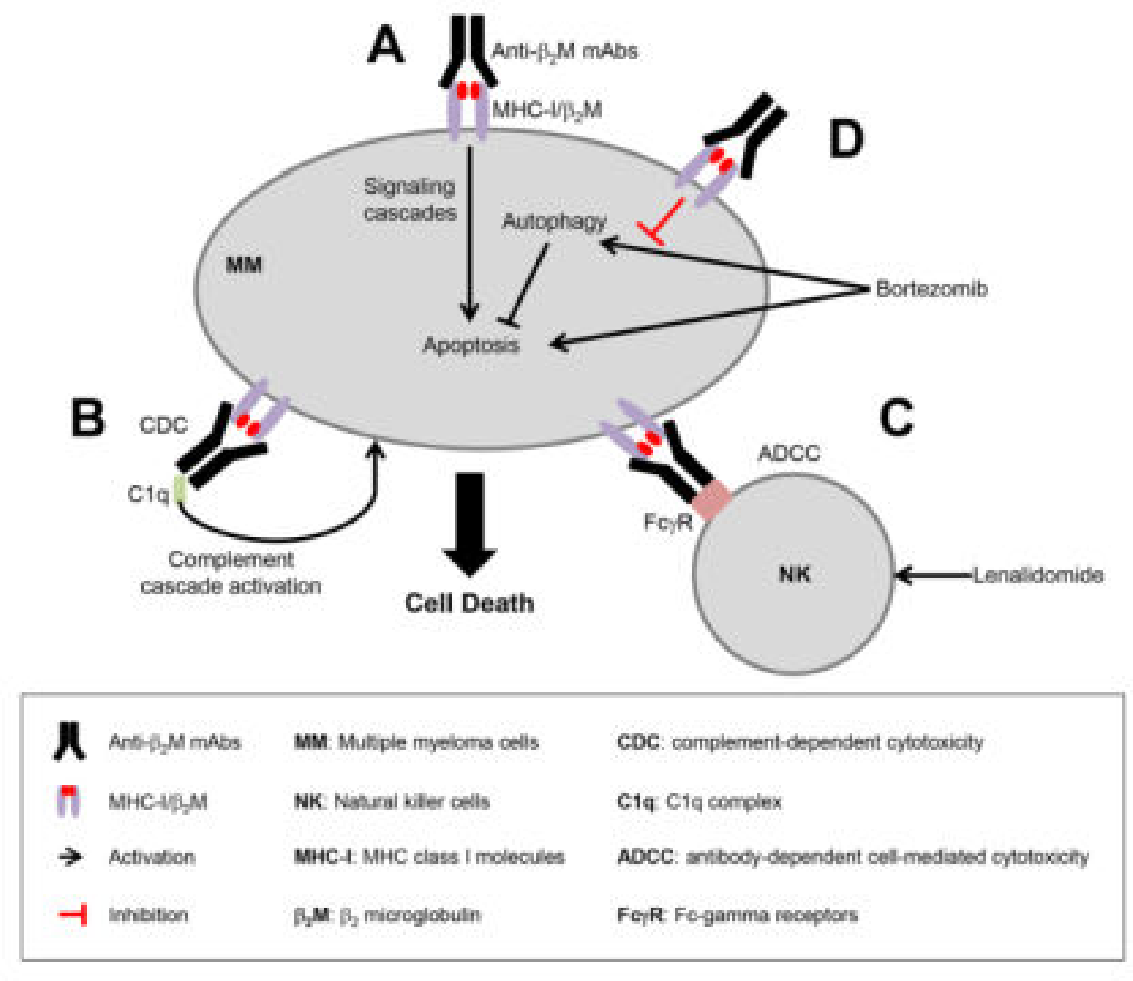
Schematic representation of the mechanistic actions of anti-β_2_M mAbs against MM cells. Anti-β_2_M mAbs induce MM cell death via (A) induction of MM cell apoptosis, and activation of (B) CDC and (C) ADCC. Lenalidomide could enhance anti-β_2_M mAb-induced ADCC activity by increasing the activity of NK cells. (D) Combination treatment of BTZ and anti-β_2_M mAbs overcomes drug resistance of BTZ by inhibiting BTZ-induced autophagy and increasing MM cell apoptosis.

Human β2M is part of major histocompatibility complex (MHC) class I molecules [9], that is involved in the presentation of peptide antigens to immune cells. Elevated β2M levels can be observed in patients with MM or other hematological malignancies, and this molecule has served as one of the key prognosis indicators in MM [10,11]. Using human-like mouse models, our research has demonstrated that anti-β2M mAbs have strong and direct apoptotic effects on MM (Figure 1A) and other hematological malignancies, with little toxicity towards normal tissues and cells [12]. The anti-β2M mAbs activate the c-Jun N-terminal kinases and inhibit extracellular-signal-regulated kinases and phosphatidylinositide 3-kinases/Akt (also known as protein kinase B). The mediated signaling pathways, and the mAbs, can recruit MHC class I molecules into and exclude receptors for growth factors, such as IL-6 and IGF-1, from lipid rafts [12,13]. Our results suggest that anti-β2M mAbs could be a novel therapeutic agent specifically targeting MM in a clinical setting.

In addition, enhancing antibody-dependent cell-mediated cytotoxicity (ADCC) and complement-dependent cytotoxicity (CDC) activities is one of the most promising ways to improve the clinical efficacy of already-approved antibodies. This concept is now actively being examined in the clinic, especially in the field of hematological malignancy treatment [14]. Our recent studies show that anti-β2M mAbs effectively lysed MM cells via ADCC and CDC (Figure 1B and 1C). We examined the anti-MM activity of anti-β2M mAbs combined with lenalidomide, an immunomodulatory drug that has been widely used in the treatment of MM [15], and we found that lenalidomide potentiated the mAb-induced ADCC activity both in vitro and in vivo against MM cells by enhancing the killing activity of natural killer cells (Figure 1C) [16]. These findings provide a rationale for combining anti-β2M mAbs with lenalidomide to improve patient outcomes in MM.

Another standard regimen to treat MM patients is proteasome inhibitor-based chemotherapy. As an example, bortezomib (BTZ) is currently being used worldwide to treat MM and mantle cell lymphoma [17]. However, adverse effects and drug resistance are emerging as great challenges for its extended application [18]. We speculated about whether the addition of anti-β2M mAb treatment would indeed improve the efficacy of BTZ alone. Our investigations showed that the combination treatment offered a much higher anti-MM effects than either agent alone, and anti-β2M mAbs enhanced BTZ-induced apoptosis in MM cells and in mouse models. Mechanistic studies showed that anti-β2M mAbs could overcome BTZ resistance by inhibiting BTZ-induced nuclear factor kappa-light-chain-enhancer of activated B cells (NF-κB) signaling and autophagy activation (Figure 1D) [19]. Thus, our studies provide a new insight in the development of anti-β2M mAbs and BTZ combination to overcome chemotherapy resistance in MM patients.

In summary, our results suggest that anti-β2M mAbs may be a more promising next-generation antibody-based immunotherapeutic agent for the treatment of MM. The clinical development of anti-β2M mAbs, both as a monotherapy or in combination with existing MM drugs, such as lenalidomide or BTZ, offers MM patients increased treatment options and improves overall patient outcome.
